# Crystal-storing histiocytosis in the stomach: A case report and review of the literature

**DOI:** 10.3389/fonc.2022.1024971

**Published:** 2022-12-15

**Authors:** Linghong Kong, Liyan Xue, Yanfeng Zhong, Shenglan Wang, Danfeng Zheng, Lining Wang, Yang Jiao, Xinpeng Zhang, Huizhong Xue, Xiaogang Liu

**Affiliations:** ^1^ Department of Pathology, Beijing Chuiyangliu hospital, Beijing, China; ^2^ Department of Pathology, National Cancer Center/National Clinical Research Center for Cancer/Cancer Hospital, Chinese Academy of Medical Sciences and Peking Union Medical College, Beijing, China; ^3^ Department of Pathology, School of Basic Medical Sciences, Peking University Health Science Center, Beijing, China

**Keywords:** crystal-storing histiocytosis, stomach, histopathology, immunoglobulin, electron microscopy

## Abstract

Crystal-storing histiocytosis (CSH) is a rare disorder characterized by the accumulation of non-neoplastic histiocytes that contain intracytoplasmic crystallized immunoglobulins. Although CSH can occur in various organs, gastric CSH is very rare. Therefore, diagnosing gastric CSH remains a challenge. Here, we present the case of a 69-year-old man with localized gastric CSH who presented with positive fecal occult blood for 2 days. Gastroscopy showed that there was a piece of irregular whitish focus in the big bend of the gastric antrum, which was soft and elastic. Histologically, the biopsied gastric mucosa showed chronic inflammation, mild activity with erosion, and numerous eosinophilic mononuclear cells containing fibrillary crystalloid inclusions in the lamina propria. Immunohistochemically, these crystal-containing cells were positive for CD68/PGM1 and Igk, which revealed that the cells were histiocytes harboring kappa light chain-restricted immunoglobulin crystals. Electron microscopic examination showed numerous high-electron-density particles in the cytoplasm of cells, with crystal structures of different sizes and shapes. This case highlights how immunohistochemistry can help with differential diagnosis and classification.

## 1 Introduction

Crystal-storing histiocytosis (CSH) is a rare lesion that results from the accumulation of immunoglobulins (Ig) in the form of crystals within tissue cells. CSH is descriptive and sounds innocuous; however, up to 90% of cases are associated with an underlying lymphoproliferative or plasma cell disease, such as multiple myeloma, lymphoplasmacytic lymphoma, or monoclonal gammaglobulinemia of unknown significance (1–3). In other words, CSH is an under-recognized paraneoplastic phenomenon, and knowledge of CSH may help detect hidden malignancies. Subtle CSH can be overlooked, while extensive CSH can mask the accompanying lymphoma. Although CSH can involve various sites, such as the bone marrow, lungs, lymph nodes, liver, spleen, gastrointestinal tract, and kidney (1, 4), it is very rare in the stomach. Therefore, diagnosing gastric CSH remains a challenge. Here, we report a case of gastric CSH and review related literature.

## 2 Case presentation

**Figure 1:**
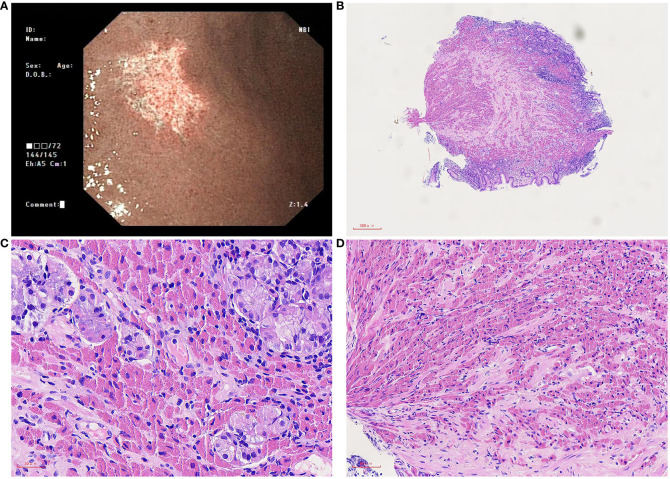
Microscopic of gastroscopy biopsy tissue. **(A)** Gastroscopy showed a piece of irregular whitish focus in the big bend of the gastric antrum. **(B)** On low power, the gastric mucosa is mildly chronic inflammation, mildly active with erosion, and a large amount of eosinophilic sub-stance deposits in the stroma, 40×. **(C)** On medium power, the inherent glands of the stomach were destroyed, a few remained glands, and a large number of tissue cells containing crystals in the stroma were infiltrated, 200×. **(D)** On high power, needle crystals in cells, 400×.

A 69-year-old man was admitted with a chief complaint of positive stool occult blood for 2 days. The patient was emaciated and anemic. The laboratory test results were as follows: white blood cell count 6.3×10^9^/L (normal: 4–10×10^9^/L), neutrophil count 3.7×10^9^/L (normal: 1.8–6.3×10^9^/L), red blood cell count 3.38×10^12^/L (normal: 4–5.5×10^12^/L), C-reactive protein 26 mg/L(normal: 0–10 mg/L), hemoglobin 88g/L (normal: 120–160 g/L), average volume of red blood cells 79.9 fL (normal: 80–100 fL), and platelet count 299×10^9^/L (normal: 100–300×10^9^/L). Gastroscopy revealed a white irregular lesion in the gastric mucosa at the big bend of the gastric antrum, which was soft and elastic (**Figure 1A**). The patient underwent gastrointestinal endoscopy, the polyps found on colonoscopy were removed, folic acid supplementation was administered, and the symptoms of anemia improved. To date, he has had no disease progression, and no lymphoproliferative disease has been found.

**Figure 2:**
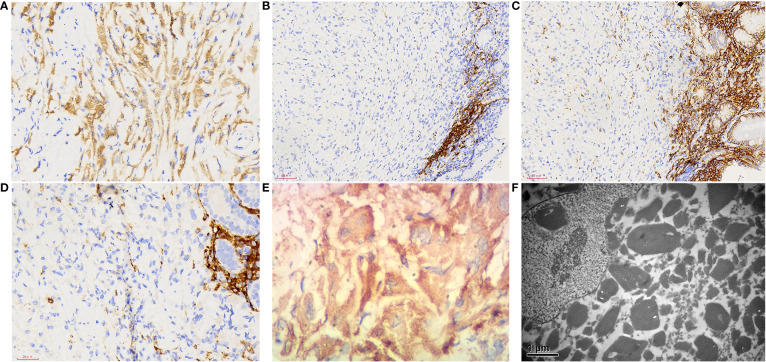
Immunophenotype and electron microscopic manifestations of gastroscopy biopsy tissue. **(A)** CD68, 400×; **(B)** CD20, 400×; **(C)** CD138, 400×; **(D)** CD79a, 400×; **(E)** kappa light chain, 400×; **(F)** Electron microscopic examination showed that a large number of high electron density particles were found in the cytoplasm of cells, with crystal structures with different sizes and shapes, 1000×.

Macroscopically, a piece of gastroscopy biopsy tissue was removed with a volume of 0.3 cm×0.3 cm×0.2 cm. Microscopic examination revealed that the biopsied gastric mucosa demonstrated chronic inflammation, mild activity with erosion, and numerous eosinophilic mononuclear cells containing fibrillary crystalloid inclusions in the lamina propria, which were composed of large polygonal and spindle histiocytes with abundant eosinophilic cytoplasm, round-to-ovoid eccentric nuclei, reticulate chromatin, and median nucleoli. Needle-shaped crystals were confined to the cytoplasm, and some were in parallel arrays (**Figures 1B–D**). Immunohistochemically, these crystal-containing cells were strongly positive for CD68/PGM1 (**Figure 2A**), but negative for CD20 (**Figure 2B**), CD138 (**Figure 2C**), CD79a (**Figure 2D**), CK (**Supplementary Figure 1A**), S-100 (**Supplementary Figure 1B**), desmin, smooth muscle actin (SMA), muscle-specific actin (MSA), and myosin. *Helicobacter pylori* (*H. pylori*, **Supplementary Figure 1C**) immunostaining was negative. Immunostaining of kappa light chain was strong and diffuse (**Figure 2E**). Immunostaining for the IgG heavy chain and λ light chain was negative. Congo red staining results were negative. Electron microscopic examination showed numerous high-electron-density particles in the cytoplasm of cells, with crystal structures with different sizes and shapes (**Figure 2F**). BIOMED-2 multiplex PCR analysis showed that immunoglobulin heavy chain (IgH-DH-JH) gene rearrangement and Igκ gene rearrangement were not detected.

## 3 Discussion

A PubMed search of the literature revealed that over 170 cases of CSH have been reported to date. However, only 17 gastric CSH (including the present case) have been described in English literature (3, 5–13). The detailed clinical and pathological findings of the patients are summarized in **Table 1**. Of these patients, 10 were men and seven were women. The mean age at diagnosis was 56 years (range, 35-86 years). There were two generalized (11.8%) and 15 localized (88.2%) types. Among them, nine patients (52.9%) were associated with or secondary to lymphoid/plasma cell neoplasm: four mucosa-associated lymphoid tissue (MALT) lymphoma with kappa restriction, one mantle cell lymphoma with lambda restriction, two diffuse large B cell lymphoma with kappa restriction, one multiple myeloma with lambda restriction, and one metachronous lymphoplasmacytic lymphoma involving the bone marrow and thymus. Five patients (31.2%) had no related diseases other than *H. Pylori* infection. During follow-up, four patients with *H. Pylori* infection had no other gastric lesions or symptoms (7–9). One patient died of an unrelated cause during the follow-up (7). Compared to other organs, gastric CSH is mainly localized, and approximately half of the cases are unrelated to clonal lymphoproliferative diseases. In contrast, they are often associated with *H. Pylori*-associated gastritis. However, in the process of reading the literature, there was one report that involved three cases (7), and their morphology was very similar to that of CSH. However, in one case the lesional cells were negative for CD68 while were positive for CD20 and CD79a. Moreover, all patients who were positive for *H. Pylori* only had anti-inflammatory treatment, and there were no other diseases during follow-up. Therefore, we only speculate that the exact diagnosis of this case may be Russell body gastritis (RBG); however, this requires further discussion.

**Table 1:** Clinical and pathological findings of previously published cases of gastric crystal-storing histiocytosis in English literature.

Year	Study	Sex/Age (yr)	Endoscopic finding	*Helicobacter pylori* infection	Associated neoplasm	Ig light chain	Follow-up
1999	Jones et al. (5)	F/35	NS	NS	Thymic lymphoma	Polyclonal	Persist
2006	Stewart et al. (7)	M/82	Gastritis	Yes	No	Lambda	Died of an unrelated cause
2006	Stewart et al. (7)	M/81	Gastritis	Yes	No	Insufficient	No symptoms or lesions
2006	Stewart et al. (7)	F/52	Erosion	Yes	No	Lambda	No symptoms or lesions
2007	Joo et al. (8)	F/56	Polyps	Yes	No	Polyclonal	No residual lesion
2013	Yano et al. (9)	F/55	Discoloration with granularity	Yes	No	Polyclonal	Alive without disease
2014	Vaid et al. (10)	M/NS	Discolored patch	NS	No	Kappa	No
2016	Kanagal-Shamanna et al. (3)	M/43	Nodule	NS	MALT lymphoma	Kappa	Alive without disease
2016	Kanagal-Shamanna et al. (3)	M/51	NS	NS	Multiple myeloma	Lambda	No
2018	Arnold et al. (6)	NS^†^	Discoloration with granularity	Yes	MALT lymphoma	Kappa	Persist
2018	Arnold et al. (6)	NS^†^	Discoloration with granularity	Yes	MALT lymphoma	Kappa	Persist
2018	Arnold et al. (6)	NS^†^	Malignant-appearing mass	No	Mantle cell lymphoma	Lambda	Died of lymphoma
2018	Arnold et al. (6)	NS^†^	Malignant-appearing mass	No	DLBCL	Kappa	Died of lymphoma
2018	Fujita et al. (12)	72/F	diffuse granular mucosa	No	No	Kappa	Alive without disease
2020	Jooet al. (11)	M/79	Ulcer, flat nodularity	No	MALT lymphoma	Kappa	No
2021	Bansal et al. (13)	M/86	Forrest IIB gastric ulcer	Yes	DLBCL	Lambda	Persist
2022	Present case	M/69	irregular whitish focus	No	No	Kappa	No symptoms or lesion

F, female; NS, not stated; M, male; MALT, mucosa-associated lymphoid tissue; DLBCL, diffuse large B cell lymphoma.

^†^Including two females and two males with ages ranging from 56 to 82 years.

Most importantly, gastric CSH needs to be differentiated from RBG, which can also show similar pathological changes. RBG is another rare entity characterized by abnormal immunoglobulin deposition in the stomach, closely related to *H. Pylori* infection (67%) (14). RBG is composed of plasma cells with small concentrated spherical immunoglobulins surrounded by endoplasmic reticulum membrane (Mott cells) (15), which is different from CSH, which consists of predominantly of histiocytes with crystallized immunoglobulin in the lysosome. RBG cases also have similar characteristics, such as frequent kappa light-chain restriction of accumulated immunoglobulin (43%) (14). There were two cases of RBG associated with lymphoplasmacytic neoplasm: one with MALT lymphoma and the other with metachronous multiple myeloma 3 years after RBG diagnosis (16, 17). However, to date, RBG has been considered a unique inflammatory reaction rather than a paraneoplastic phenomenon. Therefore, gastric CSH seems to be more significant than RBG in terms of its association with lymphoproliferative diseases. In addition, the differential diagnosis of CSH may include various diseases characterized by the aggregation of large eosinophilic tumor cells (adult rhabdomyoma, granular cell tumor, and oncocytic neoplasms) or histiocytic aggregation (Langerhans cell histiocytosis, fibrous histiocytoma, xanthogranuloma, Gaucher’s disease, and mycobacterial or fungal infection) (1–3, 6). CSH is rare and under-recognized thus may be easily misdiagnosed. In low-power image, CSH is characterized by polygonal or spindle-shaped tissue cells that contain abundant eosinophilic cytoplasm. In the high-power image, the refractile needle-like crystal substance filled the cytoplasm. Immunohistochemical analysis is helpful for differential diagnosis. In our case, immunohistochemical staining for CD68 confirmed that the large pink cells were histiocytes. Electron microscopic examination showed that numerous high-electron-density particles were found in the cytoplasm of cells, with crystal structures with different sizes and shapes, such as needles, rectangles, polygons, and diamonds. S100 protein immunohistochemical staining ruled out the possibility of a granular cell tumor and Langerhans cell histiocytosis. Congo red was used to exclude amyloidosis, and desmin, MSA, and myogenin were used to exclude adult rhabdomyoma. CK was used to rule out the possibility of metastatic cancer. The definitive diagnosis was CSH.

At present, the pathogenesis of CSH is unclear and may involve many factors, including immunoglobulin overproduction, abnormal secretion, and impaired excretion. Immunoblotting, amino acid sequencing, mass spectrometry, and gene mutation study showed that the variable region of the Ig kappa light chain was replaced by abnormal amino acids, and the sequence change led to a change in the three-dimensional structure of immunoglobulin, which promoted the formation of protein crystals and resisted the degradation of lysosomes in tissue cells, resulting in crystal accumulation. Hereditary or acquired tissue cell processing defects (processing defects) and damage of enzyme degradation of tissue cells result in the formation of Ig crystals (3, 18, 19). Among the 17 gastric CSH cases, eight were Kappa Ig light chain cases (47.1%), five were lambda (29.4%), three were polyclonal (17.6%), and one case of unknown (5.9%).

The shape, size, and staining characteristics of the crystals were relatively constant in individual cases but varied widely between cases. They can be small (2-4 μm) or large (>40 μm long), rectangular, hexagonal, rhomboid, square, elliptical, curved, or semilunar, and resemble intact or broken needles, rods, spindles, prisms, pyramids, or double pyramids (20). Crystal formation may be related to the unique structure of the secreted immunoglobulin. The finding of cytoplasmic crystal structures on electron microscope can further support the diagnosis of CSH. In our case, numerous high-electron-density particles were observed by electron microscopy in the cytoplasm of cells, showing crystal structures with different sizes and shapes, such as needles, rectangles, polygons, and diamonds.

In conclusion, we report a case of gastric CSH and summarize the clinical and pathological features of gastric CSH reported in English literature in recent years. This case highlights how immunohistochemistry can help with differential diagnosis and classification. Pathologists should know the detailed histological features of CSH to avoid misdiagnosis, and at the same time, they should be highly suspicious of the existence of associated lymphoproliferative diseases. Once the pathological diagnosis of CSH is made, it is necessary to follow up the patients for potential lymphoproliferative diseases, including detailed clinical history, serum and urine protein examination, imaging examination, and bone marrow biopsy. The treatment and prognosis of patients with CSH vary significantly depending on the associated disease.

## Data availability statement

The original contributions presented in the study are included in the article/**Supplementary Material**. Further inquiries can be directed to the corresponding author.

## Ethics statement

The studies involving human participants were reviewed and approved by Ethics Committee of Beijing Chuiyangliu Hospital. The patients/participants provided their written informed consent to participate in this study.

## Author contributions

LK, XL, LW, and YJ participated in the collection of the clinical and pathological data. XZ and HX carried out HE and immunohistochemical staining. YZ, SW, and DZ carried out electron microscopy. LX carried out PCR analysis. LK, XL, and LX evaluated the pathology of this case. LK conducted a literature search and drafted this manuscript. All authors contributed to the article and approved the submitted version.
