# Supplementary material for: Crystal-storing histiocytosis in the stomach: A case report and review of the literature
Source: Front Oncol. 2022 Dec 15;12:1024971. doi: 10.3389/fonc.2022.1024971 (PMC9798227; doi:10.3389/fonc.2022.1024971)
Supplement: Supplementary file 1 [file DataSheet_1.docx]

Supplementary Material

## Supplementary Figures


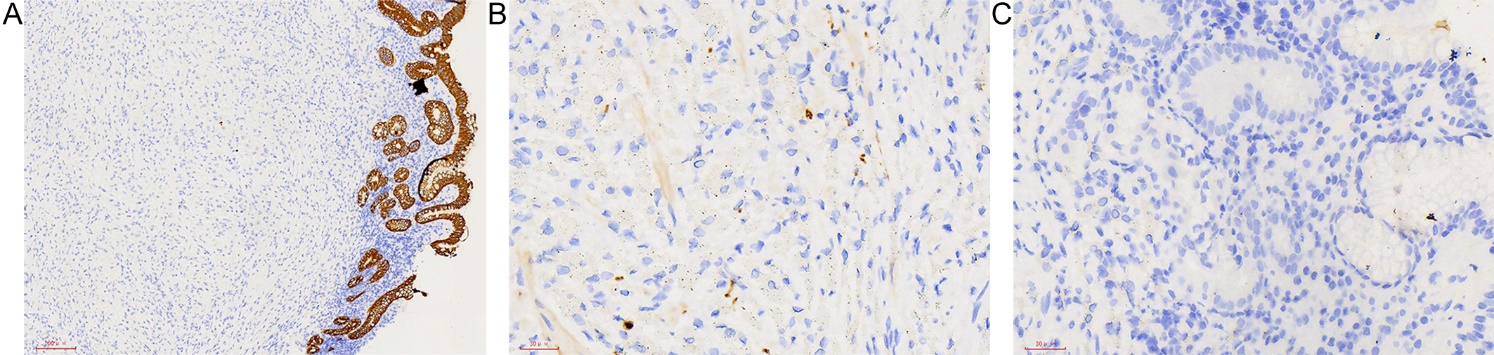


**Supplementary Figure 1.** Immunophenotype of gastroscopy biopsy tissue. (a) CK, 100×; (b) S100, 400×; (c) *Helicobacter pylori*, 400×.
